# Low incidence of adjacent segment disease after posterior lumbar interbody fusion with minimum disc distraction: A preliminary report

**DOI:** 10.1097/MD.0000000000009631

**Published:** 2018-01-12

**Authors:** Takahiro Makino, Hirotsugu Honda, Hiroyasu Fujiwara, Hideki Yoshikawa, Kazuo Yonenobu, Takashi Kaito

**Affiliations:** a Department of Orthopaedic Surgery, Osaka University Graduate School of Medicine, Suita; b Department of Orthopedic Surgery, Japan Community Health care Organization Hoshigaoka Medical Center, Hirakata; c Department of Orthopedic Surgery, National Hospital Organization Osaka Minami Medical Center, Kawachinagano; d Osaka Yukioka Medical University, Ibaraki, Japan.

**Keywords:** adjacent segment disease, cage height, minimum disc distraction, posterior lumbar interbody fusion, retrolisthesis, risk factor

## Abstract

**Study Design:**

A retrospective review of prospectively collected data.

**Objective:**

To investigate the incidence of radiographic and symptomatic adjacent segment disease (ASD) and identify possible risk factors for ASD after posterior lumbar interbody fusion (PLIF) with minimum disc distraction by selecting low-height interbody cages.

**Summary of Background Data:**

Excessive disc space distraction is reportedly 1 of the risk factors for ASD after PLIF; however, the incidence and other risk factors of ASD after PLIF with minimum disc distraction remain unclear.

**Methods:**

Forty-one consecutive patients who underwent PLIF at L4-L5 and were postoperatively followed up for a minimum of 2 years were included. The height and shape (box or bullet shape) of interbody cages was determined according to the disc height and morphology of the intervertebral space assessed on preoperative computed tomography scans to avoid excessive distraction. The incidence of radiographic and symptomatic ASD was evaluated and all demographic and radiographic parameters were compared between patients with and without ASD. Multivariate logistic regression analysis was performed to identify risk factors for ASD among the variables with *P* < .20 in univariate analysis.

**Results:**

The overall incidence of ASD was 12.2% (5/41 patients): radiographic ASD, 7.3% (3 patients); symptomatic ASD, 4.9% (2 patients). Multivariate analysis revealed preoperative retrolisthesis of L3 on extension as the sole risk factor for ASD after PLIF with minimum disc distraction (odds ratio, 2.13; 95% confidence interval, 1.00–4.05; *P* = .049).

**Conclusions:**

The incidence of ASD in this study was lower than that of ASD in our previous study about PLIF with distraction of disc space (12.2% vs. 31.8%). Minimum disc distraction by selection of low-height interbody cages is a simple and effective method to prevent ASD at the surgeons’ discretion, although preexisting retrolisthesis at the adjacent upper segment should be taken into consideration.

## 1 Introduction

Lumbar arthrodesis is commonly performed for the treatment of various lumbar pathologies. However, adjacent segment disease (ASD), which results from applying an additional significant load to the segment adjacent to the fused segment, remains a concern after lumbar arthrodesis.^[1]^ The pathology of ASD is considered to be multifactorial. According to recent reviews, several risk factors for ASD have been identified, including older age, laminectomy adjacent to a fusion segment, sagittal imbalance, preexisting facet joints and/or disc degeneration, multilevel fusion, and stopping a construct at L5.^[2–6]^ Most of these factors are not entirely avoidable.

A previous retrospective study conducted by our group found that excessive disc space distraction after posterior lumbar interbody fusion (PLIF) was a risk factor for radiographic and symptomatic ASD.^[7,8]^ Unlike the risk factors of age and anatomical characteristics, the degree of disc space distraction can easily be controlled during surgery. Based on these findings, we performed PLIF using interbody cages with a height less than that of the disc, as measured on preoperative computed tomography (CT) scans, to avoid excessive disc space distraction, as we hypothesized that minimum disc space distraction in PLIF could reduce the incidence of ASD.

The purposes of this study were to determine the incidence of radiographic and symptomatic ASD and identify possible risk factors for ASD after PLIF with minimum disc distraction by selecting low-height interbody cages in a prospective cohort.

## 2 Methods

**Figure 1:**
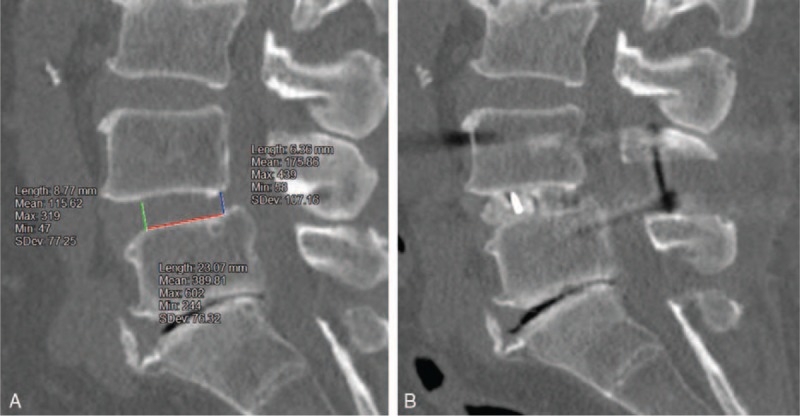
Example of selection of interbody cage height. (A) Measurement of anterior and posterior disc height on a preoperative CT scan. In this case, the anterior and posterior disc heights were 8.77 mm 6.36 mm, respectively. Thus, we selected cages with 8/6 mm (Height) × 9 mm (Width) × 21 mm (Length). (B): Postoperative CT scan. In this case, the L4-L5 vertebral height remained unchanged perioperatively (minimum disc distraction). CT = computed tomography.

This retrospective review of prospectively collected data was approved by the research ethics committee of our institution. After excluding 1 patient with brain infarction and 2 patients with postoperative progression of myelopathy because of cervical spondylotic myelopathy, the study cohort included 41 consecutive patients (14 men and 27 women; mean patient age at a time of surgery, 66.7 years; age range, 46–83 years) who underwent PLIF at L4-L5 without concomitant decompression or fusion procedures at other levels between May 2008 and July 2013, for the treatment of lumbar spinal stenosis. Each patient had at a minimum a 2-year postoperative follow-up (mean follow-up period, 41.0 months; range, 24–79 months). The indications of PLIF were spondylolisthesis with slippage greater than 3 mm and/or a posterior opening greater than 5° on dynamic lateral plain radiographs, and/or foraminal stenosis requiring total facetectomy for decompression. PLIF was performed by a single surgery team of our spine care unit through conventional open surgery (without minimally invasive surgery techniques) with total facetectomy using carbon-polyetheretherketone interbody cages and titanium alloy pedicle screws and cobalt chrome alloy rods. The height of the interbody cages were selected based on the disc height, as measured by the preoperative CT scans (sagittal images), to avoid excessive disc space distraction (minimum disc distraction, Fig. 1). To conform to the morphology of the intervertebral space, box-shaped or bullet-shaped interbody cages were selected. A bullet-shaped interbody cage was used if the disc height was <7 mm with an anterior height of 7 mm and posterior height of 5 mm. Parameters were investigated from medical records and radiographs. The radiographic parameters were measured digitally on a flat monitor at our hospital using built-in imaging software (Synapse; Fujifilm Medical Co, Ltd., Tokyo, Japan) by the author (HH), while blinded to the clinical outcomes.

### 2.1 Clinical outcome assessment

An overall clinical evaluation was made before surgery, at a maximally recovered time during the follow-up, and at the final visit at the outpatient clinic using the Japanese Orthopedic Association Score for low back pain (JOA score).^[9]^

### 2.2 Definition of ASD^[7]^

Radiographic ASD at L3-L4 was defined using plain radiographs taken before surgery and at the final visit, irrespective of the presence or absence of concomitant clinical symptoms. Radiographic ASD comprised either development of L3 antero- or retrolisthesis of more than 3 mm, a decrease in L3-L4 disc height of more than 3 mm, or intervertebral angle at flexion of < −5° (lordosis is a positive value). Clinical deterioration by L3-L4 ASD was defined as a decrease in JOA score by 4 or more points accompanied by neurological impairment in accordance with L3-L4 canal stenosis based on magnetic resonance imaging (MRI), which was postoperatively assessed every 6 months. The adjacent segment L5-S1 was not investigated in this study as degeneration at L5-S1 is frequently found preoperatively and rarely causes clinical symptoms.^[10–12]^

### 2.3 Categorizations of patients^[7]^

Patients were divided into 3 groups according to clinical and radiographic status at the time of the final visit. Group A comprised of those patients who neither had radiographic ASD nor clinical deterioration. Group B patients had radiographic ASD without clinical deterioration (radiographic ASD group). Group C patients had clinical deterioration caused by spinal stenosis at L3-L4 with or without radiographic ASD (symptomatic ASD group, including patients who underwent surgery for L3-L4 ASD).

### 2.4 Radiographic assessment^[7]^

#### 2.4.1 Fusion status

The achievement of fusion was determined by the presence of a continuous trabecular bone bridging across the disc space and the absence of screw loosening when viewed by CT scans and plain radiographs, and the absence of residual motion at the fused segment on dynamic lateral plain radiographs.

#### 2.4.2 Imaging parameters on plain radiographs

**Figure 2:**
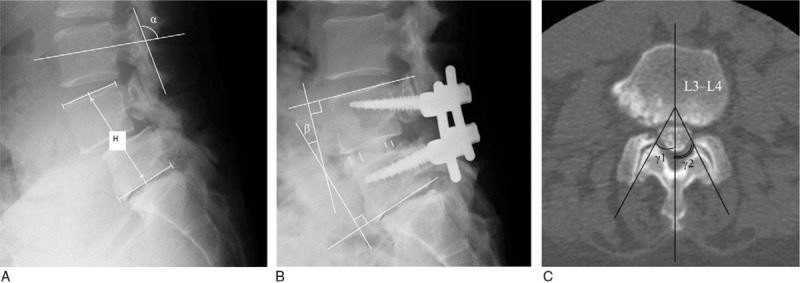
Measurement methods of the parameters on plain radiographs and computed tomography scans. (A) The L4-L5 vertebral height (H) and L3 lamina inclination (α). The L3 lamina inclination was defined as the angle formed by a line connecting the base of the superior facet joint and that of the inferior facet joint, and a horizontal line bisecting the vertebral body. (B) The L4-L5 fusion angle (β) was measured using the Cobb method. (C) γ1 is the right facet angle and γ2 is the left facet angle. Facet sagittalization and tropism were defined as (γ1 + γ2) and (γ1 − γ2), respectively.

The following parameters were measured from the preoperative plain lateral standing radiographs at L3-L4 and L4-L5: listhesis at flexion and extension (anterolisthesis is a positive value), distance of translation (|a − b|), intervertebral angle at flexion (the angle made by the endplates of the disc space; lordosis is a positive value), intervertebral angle at extension, range of motion, and disc height (between the midpoints of the upper and lower endplates). Preoperative L3 laminar inclination (Fig. 2A),^[13]^ L4-L5 fusion angle just after surgery (Fig. 2B), and lumbar lordosis between L1 and S1 before surgery (the angle made by the upper endplate of L1 and S1 in a standing neutral position) were also assessed. Cobb angles of lumbar spine were measured with the preoperative plain antero–posterior standing radiographs.

The L4-L5 vertebral height (H) was defined as the distance between the midpoint of the upper endplate of L4 and lower endplate of L5 on a lateral standing radiograph in a neutral position (Fig. 2A).^[14]^ The L4-L5 vertebral height was measured before surgery, immediately following surgery, and at the final visit (or just before surgery at the L3-L4 level to treat ASD). Parameters that related to the L4-5 vertebral height were defined as follows:

ΔH_max_ = (H just after surgery) − (H before surgery)

ΔH_final_ = (H at final visit) − (H before surgery)

#### 2.4.3 Imaging parameters on preoperative CT scans

The right and left facet angles (γ1 and γ2) were measured at the L3-L4 disc level on preoperative CT scans, and the sum of the right and left facet angles (γ1 + γ2) was defined as facet sagittalization. The difference between the right and left facet angles (|γ1 − γ2|) was defined as facet tropism (Fig. 2C).^[15]^ The degree of L3-L4 and L5-S1 facet joint degeneration was classified as grade 0 to 3 according to the grading system established by Weishaupt et al^[16]^

#### 2.4.4 Imaging parameters on preoperative MRI scans

The degree of L3-L4 and L5-S1 intervertebral disc degeneration was classified as grade 0 to 4 according to the grading system established by Pfirrmann et al^[17]^

### 2.5 Statistical analysis

Statistical analysis was performed using IBM SPSS Statistics Version 22 (IBM-SPSS, Inc., Chicago, Illinois). For univariate analysis of the risk factors for ASD, the Mann–Whitney U test was performed to compare age and radiographic parameters between Group A versus Group (B + C), and the Fisher's exact probability test was used to compare sex distributions. Multivariate stepwise logistic regression analysis was performed using variables with a probability (*P*) value of <.20 in the univariate analysis. Differences were considered statistically significant at *P* < .05.

## 3 Results

**Figure 3:**
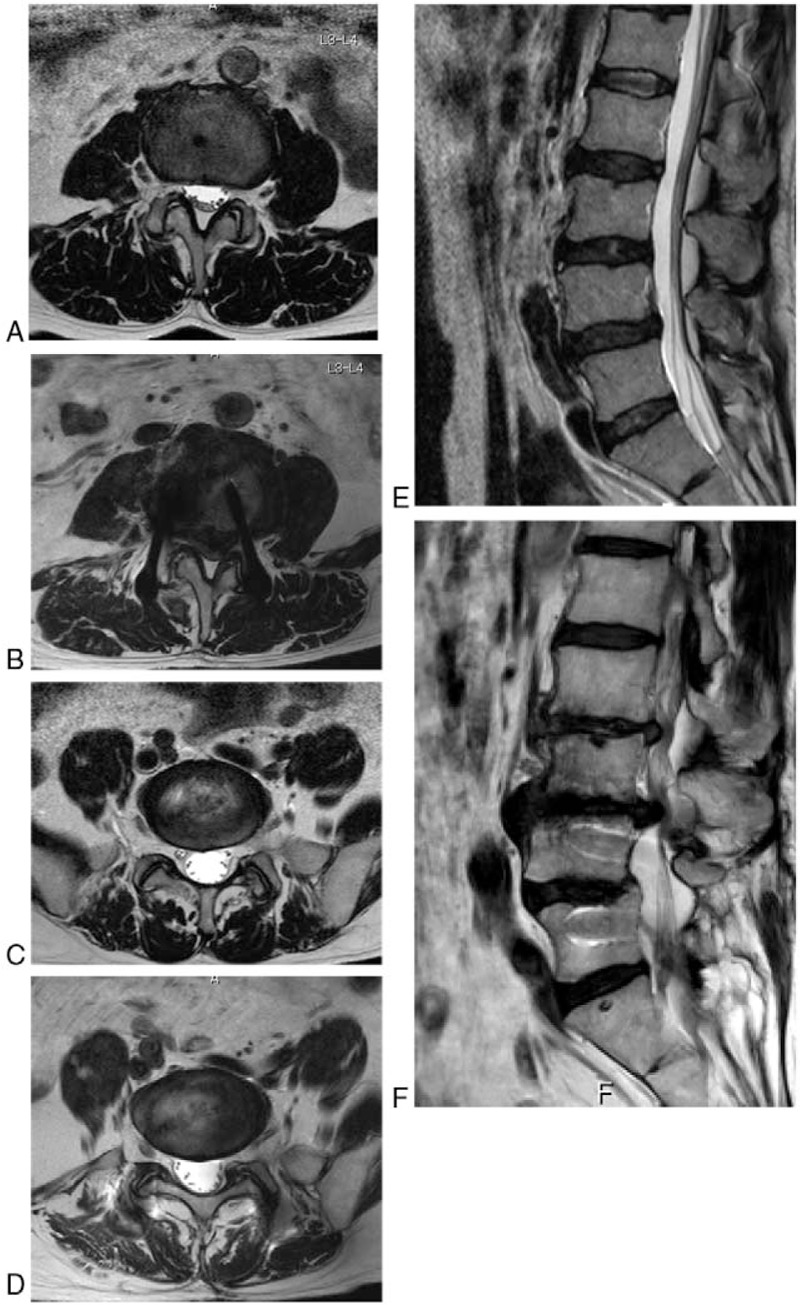
Magnetic resonance imaging scans (T2 weighted) of the patient with symptomatic adjacent segment disease at L3-L4 who underwent revision surgery 6 years after initial surgery. (A ) Axial image at L3-L4 before initial surgery. (B) Axial image at L3-L4 6 years after initial surgery. (C) Axial image at L5-S1 before initial surgery. (D) Axial image at L5-S1 6 years after initial surgery. (E) Sagittal image before initial surgery. (F) Sagittal image 6 years after initial surgery.

The overall incidence of ASD was 12.2% (5/41 patients). The incidences of radiographic and symptomatic ASD were 7.3% (3 patients, Group B) and 4.9% (2 patients, Group C), respectively. All patients in Group B had maximum postoperative JOA scores until their final visit with some radiographic findings of ASD. One patient in Group C underwent revision surgery 6 years after initial surgery (maximum JOA score, 29 points; JOA score just before revision surgery, 16 points; Fig. 3) because the spinal canal stenosis at L3-L4 progressed after initial surgery mainly due to degeneration and collapse of the intervertebral disc. Another patient in Group C received conservative treatment (maximum JOA score, 26 points; JOA score at final visit, 16 points). The patient demographic data and univariate analysis between Group A and Group (B + C) are shown in Table 1. There were no significant differences in age, sex, follow-up period, or perioperative JOA score among the groups. L4-L5 fusion without cage displacement was successfully achieved in all of the patients. No additional surgery was required for instrumentation-related complications.

**Table 1:**
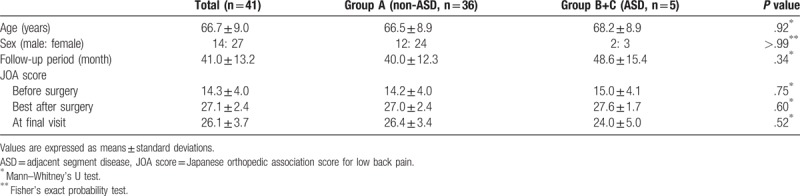
Patient demographic data and univariate analysis between Group A (non-ASD) and Group (B + C) (ASD).

Results of imaging parameters and univariate analysis of the risk factors for ASD are shown in Table 2 and Table 3. The mean ΔH_max_ and ΔH_final_, which are indexes of disc distraction upon inserting interbody cages at L4-L5, were 0.4 mm and −1.3 mm in all patients, respectively. The preoperative range of motion at L3-L4 was significantly greater in Group (B + C) than Group A (6.0° vs. 10.2°, respectively, *P* = .04). In addition to the preoperative range of motion at L3-L4, listhesis on extension at L3, distance of translation at L3, intervertebral angle at L3-L4, disc height at L3-L4, and Pfirrmann grade at L3-L4 were all identified as potential risk factors for ASD (*P* < .2 by univariate analysis). Multivariate analysis revealed that preoperative listhesis on extension (retrolisthesis) at L3 was the sole risk factor for the presence of ASD after PLIF with minimum disc distraction (odds ratio, 2.13; 95% confidence interval, 1.00–4.05; *P* = .049). Neither ΔH_max_ nor ΔH_final_ was a risk factor for ASD after PLIF with minimum disc distraction.

**Table 2:**
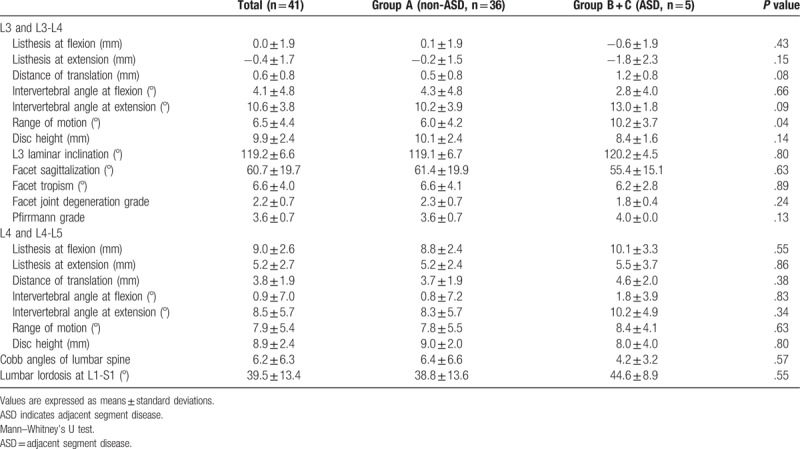
Preoperative patient imaging parameters and univariate analysis between Group A (non-ASD) and Group (B + C) (ASD).

**Table 3:**
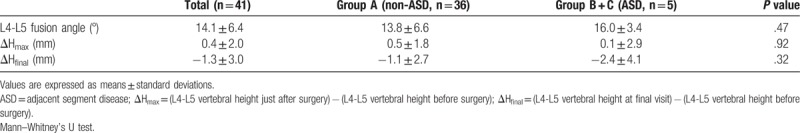
Postoperative patient imaging parameters and univariate analysis between Group A (non-ASD) and Group (B + C) (ASD).

At L5-S1, the preoperative mean facet joint degeneration grade was 1.8 ± 0.9 and 58.5% (24/41 patients) had Grade 2 or 3 of facet joint degeneration. Moreover, the preoperative mean Pfirrmann grade was 3.8 ± 0.9 and 87.8% (36/41 patients) had Grade 3 or above of disc degeneration. None of the patients had symptomatic ASD because of postoperative degeneration at L5-S1.

## 4 Discussion

In this study, with a mean follow-up period of 41 months (3.4 years), we revealed that the incidences of overall, radiographic, and symptomatic ASD after PLIF with minimum disc distraction by selecting low-height interbody cages based on preoperative CT scans were 12.2%, 7.3%, and 4.9%, respectively. Furthermore, preoperative retrolisthesis at the upper adjacent segment on extension was the sole risk factor of ASD after PLIF with minimum disc distraction. To the best of our knowledge, this is the first prospective study to investigate the incidence of ASD after PLIF with minimum disc distraction.

In lumbar interbody fusion procedures, interbody cages facilitate restoration and maintenance of disc height and local alignment. However, excessive disc space distraction by insertion of interbody cages with excessive height may increase stress in the facet joints and discs at the adjacent segments, which can result in an increased incidence of degeneration of adjacent segments.^[7,8]^ On the basis of this hypothesis, we previously conducted a retrospective study of ASD after PLIF at L4-L5 without concomitant decompression in which the measured radiographic parameters and definition of ASD were the same as in this present study.^[7]^ The results of the previous study identified ΔH_max_ as the sole risk factors for ASD after PLIF.^[7]^The mean ΔH_max_ from the previous study was 3.8 mm, which was nearly 10-fold greater than that in the present study.^[7]^ The overall incidences of ASD in our previous study were 31.8% (27/85 patients), of which 16.5% (14 patients) had radiographic ASD and 15.3% (13 patients) had symptomatic ASD.^[7]^ These incidences of ASD after PLIF, without regard to minimum disc distraction, were almost 3-fold greater than those in the present study. These findings suggest that avoiding excessive disc space using low-height interbody cages can reduce the risk of ASD after PLIF. Kawaguchi et al^[18]^ reported that the incidence of clinical ASD was 11% after lumbar laminoplasty without fusion at a mean follow-up period of 5.4 years, which was similar to the results of the present study. Interbody fusion with low-height interbody cages can reduce the incidence of adverse effects to the adjacent segments almost to the same extent as decompression without fusion.

We selected interbody cages with the same height as the disc height measured by preoperative CT scans, and the avoidance of disc space distraction attained with the disc height comparable to the preoperative height postoperatively. Although the extent of disc space distraction necessary to induce overloading the adjacent segment remains unclear, the results of this present study demonstrate that our criteria for interbody cage selection were simple and acceptable. Moreover, we selected either box-shaped or bullet-shaped interbody cages according to the morphometry of disc space, as determined by CT scans for 2 reasons: 1, because distraction of the posterior portion can occur, especially when using box-shaped interbody cages, and 2, because restoration of segmental lordosis may be an important factor in preventing ASD.^[19]^

There are several concerns about the use of low-height interbody cages. One is retropulsion of interbody cages to the spinal canal or pseudarthrosis because of instability of interbody cages. To achieve stability of interbody cages, adequate compression force should be applied to the fused segment. We confirmed the stability of the cages after applying the compression forces to the fused segment during surgery and observed no retropulsion of interbody cages in this series. Another concern is postoperative foraminal stenosis at the fused segment. However, no patients in this study demonstrated symptoms of foraminal stenosis. Resection of the cranial portion of the superior facet joint combined with total facetectomy supposedly contributed to symptom prevention.

Preoperative retrolisthesis of L3 on extension was the sole risk factors for ASD in this study. Jeon et al^[20]^ reported that there were 2 types of degenerative retrolisthesis: primary degenerative change with low pelvic incidence, and a compensatory mechanism with anterolisthesis with high pelvic incidence. Although we could not evaluate pelvic alignments in this study, most patients had anterolisthesis at L4, and so the retrolisthesis of L3 were the latter type. In this situation, L3-L4 plays a compensatory role to maintain sagittal balance and an increased load is applied to the disc and facet joints at L3-L4 preoperatively. The postoperative lack of motion at L4-L5 may require more compensatory function and apply additional load at L3-L4, which can cause ASD.

There were several limitations to this study that should be addressed. As the number of patients with ASD was relatively small in this study, we could not identify separate risk factors for radiographic and symptomatic ASD. Furthermore, several authors have reported that sagittal imbalance or mismatch of spinopelvic alignment can induce ASD after spinal arthrodesis.^[21–23]^ However, because of the lack of whole-spine radiographs in the standing position, we could not evaluate the effect of spinal alignment and global balance on ASD.

## 5 Conclusion

The incidences of radiographic and symptomatic ASD were 7.3% and 4.9%, respectively, after PLIF with minimum disc distraction. These incidences were more than half of those of our previous report on PLIF without minimum disc distraction. Selecting low-height interbody fusion cages based on preoperative CT scans to prevent excessive disc space distraction can be a simple and effective method to prevent ASD; however, preoperative retrolisthesis at the upper adjacent segment remains problematic and must be solved to obtain better outcomes.

## References

[1] Lee CK Langrana NA Lumbosacral spinal fusion. A biomechanical study. Spine (Phila Pa 1976) 1984;9:574–81. 6495027 10.1097/00007632-198409000-00007

[2] Park P Garton HJ Gala VC Adjacent segment disease after lumbar or lumbosacral fusion: review of the literature. Spine (Phila Pa 1976) 2004;29:1938–44. 15534420 10.1097/01.brs.0000137069.88904.03

[3] Malveaux WM Sharan AD Adjacent segment disease after lumbar spinal fusion: a systematic review of the current literature. Semin Spine Surg 2011;23:266–74.

[4] Lawrence BD Wang J Arnold PM Predicting the risk of adjacent segment pathology after lumbar fusion: a systematic review. Spine (Phila Pa 1976) 2012;37 (22 Suppl):S123–32. 22885827 10.1097/BRS.0b013e31826d60d8

[5] Radcliff KE Kepler CK Jakoi A Adjacent segment disease in the lumbar spine following different treatment interventions. Spine J 2013;13:1339–49. 23773433 10.1016/j.spinee.2013.03.020

[6] Zhang C Berven SH Fortin M Adjacent segment degeneration versus disease after lumbar spine fusion for degenerative pathology: a systematic review with meta-analysis of the literature. Clin Spine Surg 2016;29:21–9. 26836484 10.1097/BSD.0000000000000328

[7] Kaito T Hosono N Mukai Y Induction of early degeneration of the adjacent segment after posterior lumbar interbody fusion by excessive distraction of lumbar disc space. J Neurosurg Spine 2010;12:671–9. 20515354 10.3171/2009.12.SPINE08823

[8] Kaito T Hosono N Fuji T Disc space distraction is a potent risk factor for adjacent disc disease after PLIF. Arch Orthop Trauma Surg 2011;131:1499–507. 21706306 10.1007/s00402-011-1343-0

[9] Izumida S Inoue S Assessment of treatment for low back pain. Japanese orthopaedic association. J Jpn Orthop Assoc 1986;60:391–4.

[10] Aota Y Kumano K Hirabayashi S Postfusion instability at the adjacent segments after rigid pedicle screw fixation for degenerative lumbar spinal disorders. J Spinal Disord 1995;8:464–73. 8605420

[11] Nakai S Yoshizawa H Kobayashi S Long-term follow-up study of posterior lumbar interbody fusion. J Spinal Disord 1999;12:293–9. 10451044

[12] Cheh G Bridwell KH Lenke LG Adjacent segment disease following lumbar/thoracolumbar fusion with pedicle screw instrumentation: a minimum 5-year follow-up. Spine (Phila Pa 1976) 2007;32:2253–7. 17873819 10.1097/BRS.0b013e31814b2d8e

[13] Nagaosa Y Kikuchi S Hasue M Pathoanatomic mechanisms of degenerative spondylolisthesis. A radiographic study. Spine (Phila Pa 1976) 1998;23:1447–51. 9670395 10.1097/00007632-199807010-00004

[14] Ha KY Shin JH Kim KW The fate of anterior autogenous bone graft after anterior radical surgery with or without posterior instrumentation in the treatment of pyogenic lumbar spondylodiscitis. Spine (Phila Pa 1976) 2007;32:1856–64. 17762293 10.1097/BRS.0b013e318108b804

[15] Vanharanta H Floyd T Ohnmeiss DD The relationship of facet tropism to degenerative disc disease. Spine (Phila Pa 1976) 1993;18:1000–5. 8367766 10.1097/00007632-199306150-00008

[16] Weishaupt D Zanetti M Boos N MR imaging and CT in osteoarthritis of the lumbar facet joints. Skeletal Radiol 1999;28:215–9. 10384992 10.1007/s002560050503

[17] Pfirrmann CW Metzdorf A Zanetti M Magnetic resonance classification of lumbar intervertebral disc degeneration. Spine (Phila Pa 1976) 2001;26:1873–8. 11568697 10.1097/00007632-200109010-00011

[18] Kawaguchi Y Ishihara H Kanamori M Adjacent segment disease following expansive lumbar laminoplasty. Spine J 2007;7:273–9. 17482109 10.1016/j.spinee.2006.04.003

[19] Bae JS Lee SH Kim JS Adjacent segment degeneration after lumbar interbody fusion with percutaneous pedicle screw fixation for adult low-grade isthmic spondylolisthesis: minimum 3 years of follow-up. Neurosurgery 2010;67:1600–7. 21107190 10.1227/NEU.0b013e3181f91697

[20] Jeon CH Park JU Chung NS Degenerative retrolisthesis: is it a compensatory mechanism for sagittal imbalance? Bone Joint J 2013;95-B:1244–9. 23997140 10.1302/0301-620X.95B9.31237

[21] Senteler M Weisse B Snedeker JG Pelvic incidence-lumbar lordosis mismatch results in increased segmental joint loads in the unfused and fused lumbar spine. Eur Spine J 2014;23:1384–93. 24647596 10.1007/s00586-013-3132-7

[22] Masevnin S Ptashnikov D Michaylov D Risk factors for adjacent segment disease development after lumbar fusion. Asian Spine J 2015;9:239–44. 25901236 10.4184/asj.2015.9.2.239 PMC4404539

[23] Rothenfluh DA Mueller DA Rothenfluh E Pelvic incidence-lumbar lordosis mismatch predisposes to adjacent segment disease after lumbar spinal fusion. Eur Spine J 2015;24:1251–8. 25018033 10.1007/s00586-014-3454-0

